# Plasticity of Neuron-Glial Transmission: Equipping Glia for Long-Term Integration of Network Activity

**DOI:** 10.1155/2015/765792

**Published:** 2015-08-03

**Authors:** Wayne Croft, Katharine L. Dobson, Tomas C. Bellamy

**Affiliations:** School of Life Sciences, University of Nottingham Medical School, Nottingham NG7 2UH, UK

## Abstract

The capacity of synaptic networks to express activity-dependent changes in strength and connectivity is essential for learning and memory processes. In recent years, glial cells (most notably astrocytes) have been recognized as active participants in the modulation of synaptic transmission and synaptic plasticity, implicating these electrically nonexcitable cells in information processing in the brain. While the concept of bidirectional communication between neurons and glia and the mechanisms by which gliotransmission can modulate neuronal function are well established, less attention has been focussed on the computational potential of neuron-glial transmission itself. In particular, whether neuron-glial transmission is itself subject to activity-dependent plasticity and what the computational properties of such plasticity might be has not been explored in detail. In this review, we summarize current examples of plasticity in neuron-glial transmission, in many brain regions and neurotransmitter pathways. We argue that induction of glial plasticity typically requires repetitive neuronal firing over long time periods (minutes-hours) rather than the short-lived, stereotyped trigger typical of canonical long-term potentiation. We speculate that this equips glia with a mechanism for monitoring average firing rates in the synaptic network, which is suited to the longer term roles proposed for astrocytes in neurophysiology.

## 1. Plasticity as the Cellular Basis of Learning and Memory in the Central Nervous System

At a high level of abstraction, the brain is essentially an organ that detects environmental stimuli, processes the received sensory information, and initiates an appropriate motor response. From this perspective, the primary role of the brain is information processing, and the computational processes associated with transforming input to output are centred on the network of trillions of synapses through which the signals are relayed. The train of action potentials initiated in sensory neurons must be transduced by the central synaptic networks in such a way as to reliably trigger a pattern of action potentials in the motor neurons that effect the necessary coordinated activation of muscles needed to evoke a behavioural response. It is thus widely accepted that, despite defying human comprehension, there must be a particular spatiotemporal pattern of network activity reliably associated with generating a given response to a given external cue.

To cope with a complex and changing environment, the synaptic network must also be adaptable, such that experience can refine and reorganize the spatiotemporal patterns of network activity in response to, for example, injurious stimuli. This adaptability requires controlled alteration of synaptic strength, a phenomenon termed synaptic plasticity [1].

The forms and mechanisms of synaptic plasticity have been extensively studied for many decades in many brain regions [2–6] and can range in time from short-term changes that last for seconds [7] to long-term changes that can last for months or longer [8]. Common functional requirements for synaptic plasticity are coordinated activation of presynaptic and postsynaptic cells (associativity), close temporal association of activity (coincidence detection), and induction by patterns of action potentials occurring at defined synapses (input specificity). With these concepts, many features of learning and memory processes observed at the organismal level can be understood as arising from underlying cellular processes.

Over the last few decades, the view of glial cells in the brain has developed from passive, homeostatic components to active signalling elements. Unsurprisingly, much of the evidence supporting a computational role for astroglia comes from the effects of astrocyte signalling on synaptic transmission and synaptic plasticity; clearly, if astroglia are able to modulate synaptic plasticity, then they are functionally implicated in information processing. Less attention has been focussed on a tangential question: can astrocyte signalling networks themselves exhibit activity-dependent changes in strength? Do pathways for neuron-glial transmission also vary in connectivity and strength in response to defined patterns of activity; do astrocytes exhibit plasticity that could allow them to directly mediate encoding of memory processes?

In this review, we summarize the current evidence for plasticity in neuron-glial transmission (with an emphasis on astroglia), the different forms that this plasticity can take and speculate on the potential computational properties of known forms of glial plasticity. We argue that neuron-glial plasticity has several strikingly different features from synaptic plasticity, which are better suited to the temporal scale over which astrocyte calcium signals operate and the neurophysiological roles in which glia are implicated.

## 2. The Discovery of Neuron-Glial Transmission

As electrically passive cells, astrocytes were once thought to depolarize solely as a result of the changes in extracellular potassium concentration associated with neuronal activity, reflecting a passive potassium conductance. This view was overturned by experiments performed in neuron-free astrocyte cultures, in which direct depolarization in response to excitatory and inhibitory neurotransmitters were recorded, demonstrating that astrocytes expressed ionotropic neurotransmitter receptors [9, 10]. These discoveries raised an obvious question: what would be the benefit of ionotropic receptors in nonexcitable cells? They also stimulated more focussed attention on the potential for glial cells to play more active roles in neurophysiology.

The next key advance in understanding of neuron-glial transmission was the discovery of metabotropic receptors linked to second messenger signalling pathways. An important first step was the use of astrocyte-enriched cultures to demonstrate turnover of radiolabelled inositol phospholipids in response to acetylcholine or noradrenaline administration [11], indicating that these neurotransmitters could stimulate inositol phospholipid metabolism within the astrocyte membrane. These results coincided with the publication of evidence implicating inositol phosphates in the regulation of calcium signalling [12–14], and, coupled with the knowledge that neurons in culture display calcium oscillations in response to neurotransmitters [15, 16], this prompted investigations into calcium signalling in astrocytes.

In 1990, Cornell-Bell et al. [17] cultured astrocytes from rat hippocampus, loaded them with the calcium-sensitive dye fluo-3, and recorded the responses evoked by extracellular glutamate. This landmark publication yielded several fundamental discoveries about astrocyte calcium signalling, the first of which was the heterogeneous nature of the astrocyte response to sustained glutamate application. Cell responses were categorized as sustained oscillations, damped oscillations, or step-responses. This heterogeneity indicated both the diverse functionality of astrocyte responses to neurotransmitter application, as well as identifying apparent subpopulations with differing ligand sensitivities. Secondly, calcium waves generated by glutamate treatment were shown to propagate from cell to cell, demonstrating network-wide signalling. Finally, by repeating the experiments in calcium-free media, the authors determined that the majority of the glutamate response was the result of calcium being liberated from intracellular stores [17].

In addition to heterogeneity within cultures from individual brain regions, astrocyte cultures obtained from the cortex or cerebellum displayed different response characteristics to those observed in hippocampal cells. The glutamate analogue quisqualate produced calcium responses in approximately one-quarter of cerebellar and cortical astrocytes, but less than one-tenth of hippocampal cells responded to this treatment [18], suggestive of differential glutamate receptor expression in these populations. This study also demonstrated direct calcium influx through ionotropic AMPA receptors, providing a signalling rationale for ion channel expression in nonexcitable cells [18].

These key discoveries from cultured astrocytes, bolstered by many other demonstrations of calcium-evoked responses to neurotransmitters (see [19]), established the concept that glial cells were capable of detecting extracellular stimuli, and transducing the signal into an intracellular response, implicating glia as potential signalling agents in neurophysiology. The next major advance was to extend the investigations of glial signalling into* in situ* tissue slice preparations where synaptic release could be directly triggered experimentally.

The first demonstration of astrocyte calcium signalling in response to electrical stimulation of neurons came from the hippocampus [20]. Hippocampal dentate gyrus neurons project glutamatergic mossy fibres to the CA3 region, and so mossy fibre stimulation was used to investigate CA3 astrocyte responses to glutamate release in slices preloaded with the calcium indicator, fluo-3. Under these conditions calcium waves were observed in two cellular populations, an initial neuronal response, which was followed by a kinetically distinct astrocyte response. As with cultured astrocyte experiments [17], calcium waves observed in this preparation were both intracellular and intercellular, strengthening the evidence for communication occurring via calcium signal propagation throughout the glial syncytium.

Subsequent investigations confirmed activity-dependent astroglial calcium responses in neuromuscular junction [21], retina [22], hippocampal CA1 region [23], cerebellar Bergmann glia [24], thalamus [25], and cortex [26]. In the decades since, astrocytes have been shown to respond to a vast range of transmitters and other signalling molecules, throughout the CNS. This includes (but is not limited to) monoaminergic neurotransmitters (noradrenaline, serotonin, histamine, and dopamine), neuropeptides (neuropeptide Y and substance P), ATP, acetylcholine, GABA, nitric oxide, and endocannabinoids [19, 27–31]. This period also saw the study of neuron-glia signalling extend beyond astrocytes into microglia [32], oligodendrocytes [33], and other cells in the glial lineage [34, 35].

The final step in confirming neuron-glial transmission as a fundamental aspect of neurophysiology was demonstrating glial responses to sensory input. The* ex vivo* rat retina preparation is a model system to which a natural stimulus—light—can be applied in a controlled manner, to examine cellular events in intact networks. Constant illumination triggered calcium transients in Müller cells (the specialized glial cell of the retina), and flickering light increased the frequency of the calcium oscillations in the glia [36]. The generation of light-evoked glial calcium signals depended on neuronal firing, providing strong evidence for a neuron-glial signalling route activated by natural, sensory stimulation of neurons* in situ*.

Calcium imaging studies of astrocyte responses to sensory stimuli have also been performed* in vivo*. Cortical astrocytes were shown to exhibit spontaneous calcium signals, which correlated with neuronal firing frequency, and spread through astrocyte networks [37]. The barrel cortex, part of the somatosensory cortex, is a specialized brain region found in some rodents that receives inputs (via the thalamus) from the whiskers. Whisker stimulation triggered calcium increases in astrocyte processes and soma, which were delayed relative to increases in local field potential [38]. Similar findings were obtained by measuring somatosensory cortex activity during limb movement, in which astrocyte calcium oscillations exhibited input-specific response characteristics [39] indicating that astrocytes in this brain region are not only responsive to external stimuli but also engaged in a selective manner. Cerebellar Bergmann glia detect and respond to motor activity with calcium responses that vary in range from subcellular microdomains to syncytial waves that spread through the cerebellar cortex [40].

This body of work has shown that, in common with neuronal transmission, the modes of neuron-glial communication vary in their anatomy, molecular mechanisms, and spatiotemporal kinetics. In some contexts, specialized terminals between neurons and glia are known [41, 42], with vesicular release targeting juxtaposed receptors on glial cell membranes. In other contexts, neurotransmitter release acts by diffusion to extrasynaptic receptors on glia, analogous to volume transmission mechanisms of neuron-neuron communication [43]. For the purposes of this review, we are collectively describing these different forms of cell to cell communication as “neuron-glial transmission” to encompass the different release sites, transmitters, and glial cell types in which plasticity has been described.

The concept of neuron-glial transmission as a ubiquitous feature of neurophysiology is now widely accepted [44]. The physiological roles of this ancillary signalling network, linked to the synaptic network but with markedly different spatiotemporal properties to action potential propagation, are still a matter of lively debate [45]. One of the major consequences of neuron-glial signalling is the calcium-dependent vesicular exocytosis of gliotransmitters, which gives feedback to modulate the synaptic network. This concept has been extensively reviewed elsewhere [46, 47], but a concept that has received less attention is the capacity for bidirectional communication between synaptic and glial networks to be altered in an activity-dependent manner. The ability to encode long-lasting changes in strength linked to the pattern of incident activity received is a central requirement for learning and memory. In the following sections, we present evidence for the existence of such glial plasticity in several distinct brain regions, utilizing several different receptor signalling pathways. Our case is that glia can exhibit plasticity, but of notably different character to synaptic plasticity.

## 3. Metabotropic Glutamate and Muscarinic Acetylcholine Receptor Signalling in Hippocampus

One of the earliest reports of plasticity in glial cell signalling was related to metabotropic glutamate receptor (mGluR) evoked calcium signalling in cultured visual cortex astrocytes [48]. Repetitive stimulation of astrocytes with glutamate, with recovery periods of 2 to 60 min between applications, led to a progressive increase in the frequency of calcium oscillations for the later treatments. This “priming,” or sensitization, of astrocytes to subsequent glutamate stimuli could be blocked by inhibitors of NO synthase but recovered by coapplication of NO donors [48]. The implication is that upregulation of NO synthesis caused a lasting potentiation of the calcium response to mGluR activation.

This initial discovery was followed by an* in situ* investigation in hippocampal slices [49]. Here it was demonstrated that repetitive stimulation with the mGluR agonist ACPD was sufficient to reproduce the priming effect observed for glutamate. Priming was most prominent in astrocytes that initially responded with a low frequency of calcium oscillation, suggesting that there is a ceiling effect where potentiation is saturated at an upper level, and pretreated cells exhibit heterogeneity in their initial rate of oscillation. Importantly, this study also demonstrated that the potentiation could be evoked by synaptic stimulation. Activation of mGluR-evoked calcium responses in astrocytes was triggered in response to stimulation of neuronal afferents (Schaeffer collaterals), and the frequency of calcium spikes in astrocytes was increased by increasing stimulus intensity or frequency. Such activity-dependent stimulation showed similar priming capacity: later stimulation under the same conditions showed higher-frequency calcium oscillations in astrocytes. Electrical stimulation also potentiated the responses to applied ACPD, and this potentiation could last for at least 3 hours [49]. Collectively, these studies showed that the calcium response to glutamate in astrocytes could be potentiated in a lasting fashion—a defining characteristic for plasticity.

The detailed mechanism for priming has not been determined, but the intracellular signalling network that regulates mGluR5-evoked calcium oscillations in astrocytes has been characterized in detail [50–52]. The frequency of calcium oscillations increases with glutamate concentration, suggesting frequency-modulation as a means of signal encoding [53]. Oscillation mechanism itself depends on cyclical phosphorylation and dephosphorylation of amino acid residues in the mGluR5 structure that mediates interaction with the heterotrimeric G_q/11_ protein; so called dynamic uncoupling [50, 54]. The oscillation frequency can therefore be modulated by alteration of kinase and phosphatase activity, by pharmacological modulators and by variation in the density of mGluR5 in the plasma membrane [51]. These mechanisms suggest that crosstalk between signalling pathways that regulate kinase/phosphatase balance or expression levels of mGluR5 could underlie modifications in the frequency of calcium response to glutamate release [55].

In addition to mGluR5 activation following Schaeffer collateral stimulation, Perea and Araque [56] reported astrocyte calcium signalling evoked by stimulation of the alveus input. Despite alvear terminals releasing glutamate, the target for transmitter released from this input was muscarinic acetylcholine receptors (mAChR). Thus, cholinergic transmission is also able to stimulate calcium signalling in hippocampal astrocytes. Also of note (from the perspective of plasticity), these authors went on to show that crosstalk between the alvear and Schaeffer collateral inputs was nonlinear [56]. Calcium mobilization in response to costimulation did not simply sum, despite the fact that the astrocyte calcium signalling apparatus was neither saturated nor exhausted. Indeed, the interaction between mAChRs and mGluRs showed complex frequency dependence. At low stimulation frequencies, potentiation of calcium responses was observed for costimulation, whereas at higher frequencies, the potentiation switched to depression. Antagonism of either receptor class eliminated the crosstalk effects, suggesting that computation was occurring at the level of the astrocyte signalling networks.

Honsek et al. [57] took a different approach and investigated whether stimulation paradigms known to effectively evoke long-term plasticity in the synaptic network had similar effects on neuron-glial transmission. In hippocampal slices, the authors showed that the amplitude of astrocyte calcium signals was dependent on the number of Schaeffer collateral synapses activated, but that induction of either long-term potentiation (LTP) or long-term depression (LTD) in the adjacent CA1 neuron synapses had no detectable effect on the magnitude of astrocyte calcium responses. This suggests that the induction mechanisms (and computational rules) governing glial plasticity are distinct from those governing synaptic plasticity.

Xie et al. [58] have also investigated the effects of long-term changes in neuronal firing rate in hippocampal slices on astrocyte mGluR responses. Suppression of firing with TTX altered astrocyte calcium kinetics in a manner consistent with upregulation of mGluRs. The reciprocal effect was seen when slices were incubated in raised K^+^ concentrations (5 mM) to increase firing rate. This caused a decrease in the responsiveness of astrocytes to mGluR agonists, suggesting bidirectional changes in the strength of mGluR-evoked calcium signals depending on the rate of neuronal firing. This plasticity in mGluR response was also selective, as responsiveness to activation of other G_q_ coupled receptors was unaffected [58].

Collectively, these studies demonstrate that the strength of neuron-glial transmission via activation of mGluR and mAChR can vary in response to alterations in neuronal firing rates, but these changes are initiated by different patterns of activity to those that trigger synaptic plasticity.

## 4. Ectopic Transmission to Bergmann Glia

Bergmann glial cells are astroglia of the cerebellar cortex with a characteristic morphology [59]. Radial fibres project from the cell soma, from which sprout multiple lateral projections known as microdomains. These microdomains enclose the synapses of the molecular layer, positioning glial membranes in the near vicinity of sites of transmitter release.

Cultured Bergmann glia were first shown to express calcium-permeable AMPA receptors in 1992 [60], and soon after pharmacological activation of these receptors* in situ* (in acutely isolated slices) was confirmed [61]. In 1997, two groups demonstrated activity-dependent activation of AMPA receptors after stimulation of the climbing fibre [62] and parallel fibre [63] inputs to Purkinje neurons, along with currents linked to electrogenic uptake of glutamate by EAAT1 and EAAT2 transporters. It was initially assumed that the glial AMPARs were engaged by glutamate escaping from the synaptic cleft by diffusion, but further investigation revealed a more specialized form of transmission.

Glial AMPAR currents were found to show rapid kinetics consistent with local vesicular delivery, rather than diffusion of glutamate from distant sites [41, 64]. Significantly, unitary release events could be detected in the glia, and these events were asynchronous with unitary responses in the adjacent Purkinje neuron, indicating the cells were detecting vesicular release from different presynaptic sites [65]. Bergmann glia lack direct synaptic connections with climbing and parallel fibres, but anatomical and pharmacological evidence is consistent with vesicular release from regions of the terminals distant from the active zone to activate AMPAR on the opposed glial membrane [41]. This phenomenon has been termed ectopic release [66].

Ectopic transmission to Bergmann glia exhibits both short-term plasticity and long-term plasticity. Short-term plasticity mirrors the plasticity observed at the adjacent synapses—paired pulse depression at climbing fibres, and paired pulse facilitation at parallel fibres—but in both cases the magnitude of plasticity is greater at Bergmann glia than Purkinje neurons [64, 67]. Facilitation at parallel fibre synapses peaks at around a 2-fold increase in the amplitude of the second pulse, which is sustained during trains of high-frequency stimulation. In contrast, initial facilitation of glial currents reaches 5-fold enhancement, but tetanic stimulation leads to rapid loss of transmission [67].

At climbing fibres, the opposite phenomenon, paired pulse depression, reduces AMPAR currents by around half. For ectopic transmission to glia, depression is more pronounced, with >90% of AMPAR current being lost [64, 68]. The pattern of depression against interpulse interval also differs for ectopic transmission, especially when release probability is reduced by decreasing extracellular calcium concentration. Rather than a progressive recovery of amplitude with increasing pulse interval, ectopic sites show a biphasic time course with a trough at 100 ms interval [64].

While short-term plasticity resembles the synaptic pattern in many respects, long-term plasticity is strikingly distinct. Many forms of LTP and LTD are known at parallel and climbing fibre synapses with both presynaptic and postsynaptic mechanisms [69, 70]. In contrast, ectopic transmission is dominated by long-term depression [71]. Stimulation of either input to the glial cell at frequencies greater than ~0.1 Hz causes a progressive reduction in AMPAR current amplitude, which persists after returning to lower baseline frequencies. This LTD of ectopic transmission is input specific [68] and the mechanism for depression is depletion of presynaptic release competent vesicles [72]. Ectopic sites appear to lack the fast vesicle recycling mechanisms present at the active zone, resulting in slow recovery after exhaustion of the readily-releasable pool [72]. The computational consequence of this is that ectopic transmission to glia is inversely proportional to the average firing rate of parallel and climbing fibres.

## 5. Synaptic Transmission to NG2 Cells

The mode of neuron-glial plasticity that most closely resembles synaptic plasticity is found in NG2 cells. These cells have been variously described as oligodendrocyte precursor cells, polydendrocytes, synantocytes, and NG2 cells, and their precise nature or subclassifications is still an active area of debate [34]. These cells receive direct connections from neuronal terminals closely akin to synapses, although uncertainty still exists as to their functional status [42].

Postsynaptic currents in NG2 cells showed similar short-term plasticity (paired pulse facilitation) to adjacent Schaeffer collateral to CA1 pyramidal neuron synapses in hippocampal slices [73]. In contrast to hippocampal astrocyte mGluR responses [57], and ectopic transmission to Bergmann glia [72], theta burst stimulation of Schaeffer collaterals reliably evoked LTP in NG2 cell synapses, similar to the LTP evoked at neuronal synapses [73]. Although the pattern of stimulation required to evoke NG2 LTP was similar to neuronal forms of LTP, the mechanism of LTP induction and maintenance differed in the glia. NG2 cell LTP did not depend on NMDA receptor activation; instead, it depended on calcium influx through a calcium-permeable isoform of AMPA receptor. Buffering internal calcium with the chelator BAPTA reversed LTP to LTD, suggesting a bidirectional form of plasticity dependent on the amplitude of intracellular calcium concentration, as observed at neuronal synapses [73].

The mechanisms of NG2 cell plasticity have since been investigated in more detail in other regions—cerebellum and optic nerve [74]. The levels of calcium-permeable AMPA receptors present in NG2 postsynaptic sites were shown to increase on activation of mGluRs, in parallel with PI3K, PICK-1, and JNK signalling. In contrast, activation of purinergic receptors decreased AMPAR density, again demonstrating bidirectional plasticity in expression levels and the strength of transmission in these cells.

## 6. Neuromuscular Junction

The neuromuscular junction (NMJ) is surrounded by perisynaptic Schwann cells (PSCs), which are nonmyelinating glia that functionally resemble astrocytes of the central nervous system [75]. Todd et al. [76] investigated how patterns of stimulation known to evoke plasticity in NMJ transmission were detected and encoded into calcium responses by PSCs. Continuous high-frequency stimulation (at 20 Hz) evoked a posttetanic potentiation of NMJ transmission, and generated one or two high amplitude calcium responses in PSCs. In contrast, stimulating the presynaptic fibres with intermittent bursts (but with the same overall number and frequency of stimuli), resulted in posttetanic depression of transmission, and lower amplitude oscillations in glial calcium [76]. This shows that, as with hippocampal neuron-glial transmission, the evoked PSC calcium response at the NMJ can offer a means of discrimination between different patterns of synaptic transmission.

The plasticity of the calcium responses in PSCs has also been explored, by suppression of synaptic transmission with *α*-bungarotoxin and by chronic stimulation of the nerve [77]. The results showed complex changes in calcium responses that did not correlate in a straightforward manner with associated changes in synaptic strength and short-term plasticity. Suppression of transmission caused decreases in amplitude and time to peak for calcium responses evoked by subsequent nerve stimulation, whereas chronic stimulation had no obvious effect on evoked PSC calcium signals. Exogenous application of ATP and muscarine, however, revealed that bungarotoxin treatment prolonged calcium responses, while chronic stimulation altered PSC sensitivity to the exogenous agonists, increasing sensitivity to ATP but decreasing sensitivity to muscarine [77]. Collectively, these results suggest complex changes in receptor expression level and calcium signalling kinetics in PSCs as a result of manipulating neuronal firing rates.

Recently, this capacity for PSCs to utilize calcium responses as a mechanism for detecting differences in synaptic strength at the NMJ has been proposed as a means for influencing selection between competing presynaptic terminals during development [78].

## 7. Crosstalk and Gliomodulation

Another mechanism for plasticity, which can be viewed as distinct from frequency-evoked changes in transmission strength, is the modulation of transmission by concurrent activation of parallel signalling pathways. Crosstalk between pathways is well known in synaptic transmission, through activation of neuromodulatory receptors coupled to second messenger signalling cascades, with the classic examples being monoamine transmitters such as adrenaline, noradrenaline, 5-HT, and dopamine [79]. Similarly, purines, nitric oxide, and endocannabinoids mediate local and global modulation of synaptic transmission. Such neuromodulation plays multiple roles in information processing [80].

Interpretation of the effects of neuromodulators on astrocyte calcium signalling is complex, however, because many of the physiological receptor agonists can directly stimulate calcium responses in the astroglia. Nevertheless, evidence is accumulating that in addition to direct effects, coapplication of several neuromodulators with other transmitters generates different calcium responses in the glia compared to transmitter alone.

Nitric oxide can directly activate calcium influx in astrocytes at high concentrations [81, 82], but at lower (more physiological) concentrations it appears to modulate other signalling pathways. In cultured astrocytes stimulated mechanically, NO promoted the speed and range of calcium waves propagating through coupled glia [81, 83] and accelerated refilling of calcium stores [84]. Similarly, NO increased the frequency of spontaneous calcium oscillations in astrocytes in brain slices incubated at physiological temperatures, which was shown to reduce oscillation frequency compared to incubation at room temperature [85]. These results suggest that NO is able to modify the intrinsic calcium loading and release apparatus at some level, modulating the cells' sensitivity to other calcium-linked transmitters, and may account for the proposed role of NO in priming of hippocampal mGluR responses (see above [49]).

Adenosine has also been shown to mediate complex crosstalk with calcium signalling pathways in astrocytes. A1 receptor activation potentiates both mGluR [86] and mAChR [87] triggered calcium responses in cultured astrocytes. Adenosine similarly potentiated responses to ATP in cerebellar astrocytes, but via A2B receptor activation [88]. In contrast to potentiation of the peak of ATP responses by A2B receptors, another study showed that A1 activation depressed the sustained calcium elevation that followed P2YR responses [89]. The underlying mechanisms linking P1 receptor activation to P2 responses appear to differ in cerebellar and cortical astrocytes, but the consistent effect is a sensitization of cells to ATP and glutamate as calcium-mobilizing agonists. In the intact retina, incubation with adenosine increased the frequency of light-evoked calcium transients in Müller cells [36], suggesting that crosstalk between these pathways is not restricted to cultured cells.

One of the most intriguing examples of complex crosstalk in astrocyte responses is the effect of noradrenaline acting through *α*1 receptors. Many studies have shown direct activation of calcium responses by *α*1R activation, in cultures,* ex vivo* slices, and* in vivo* [28, 90, 91]. However, in addition to this direct route for neuron-glial transmission, *α*1R activation also causes modulation of responses to other calcium mobilizing agonists. In cultured cells, coapplication of the *α*1 agonist phenylephrine with the mGluR1 agonist DHPG dramatically suppressed calcium oscillations (and associated glutamate release) that were evoked by DHPG alone [28]. In contrast to these results, astrocytes in the supraoptic nucleus (SON) exhibit synergistic responses to coapplication of ATP and phenylephrine [92]. The potentiation evoked by costimulation in SON appears to be mediated by both *α*- and *β*-adrenoceptors, with *β* receptor antagonists blocking the calcium responses evoked by phenylephrine and ATP, suggesting a tonic, permissive effect of *β* receptor activity.

Similar complex adrenoceptor interactions have also been reported* in vivo*. In awake mice, wide ranging calcium signals recorded in cortical astrocytes appear to predominantly arise from noradrenergic transmission from locus coeruleus projections [91, 93]. This is in contrast to* ex vivo* preparations or anaesthetized animals, where local excitatory network activity engages mGluRs in astrocytes, suggesting distinct modes of operation linking sensory input to glial calcium responses depending on level of consciousness. Similar NA-evoked astrocyte responses have been described in cerebellar Bergmann glia and visual cortex astrocytes in awake mice [94], suggesting that volume transmission from locus coeruleus is a widespread mechanism for coupling arousal states to astrocyte calcium signals. However, Paukert et al. [94] also showed that local network activity could be detected by glia and that NA release associated with arousal synergized with excitatory inputs due to light stimulation in the visual cortex, in a manner reminiscent of results in SON astrocytes [92].

Collectively, these studies demonstrate that the responsiveness of astrocytes to numerous neurotransmitters could be altered, both positively and negatively, by coincident activation of modulatory receptors. This represents a form of gain modulation of glial responses, akin to that observed in synaptic networks.

## 8. Other Forms of Glial Plasticity

We have so far focussed on forms of plasticity in neuron-glial transmission linked to receptor-mediated calcium signalling pathways, on the assumption that these mechanisms represent an analogue for synaptic transmission. However, several other forms of plasticity in neuron-glial connectivity have also been reported which could impact on signal conditioning.

In addition to receptor-mediated signalling, many neurotransmitters also generate inward currents in astrocytes through electrogenic uptake. While this phenomenon is clearly important for termination of synaptic transmission, there is also evidence that the Na^+^ influx associated with uptake may have a signalling role [95]. Several reports have demonstrated both potentiation and depression of transporter currents in astrocytes. Glutamate transporter currents in cultured cerebellar astrocytes mirrored LTP of granule neuron-Purkinje neuron synapses [96]. Similarly, hippocampal astrocytes also show increased uptake in response to LTP in adjacent synapses [97, 98]. In contrast, Bergmann glial transporter currents in cerebellar slices exhibit long-term depression that mirrors glial AMPAR LTD [71]. In the dorsal horn of the spinal cord, glutamate transporter currents in glia evoked by afferent stimulation showed frequency-dependent depression at low frequencies [99], which was reminiscent of Bergmann glial depression.

Another form of plasticity that could impact on information processing in glial networks is the modulation of gap-junctional coupling between astroglial cells. Both short-term and long-term modulation of connexin function and expression by neuronal activity has been reported in both cultured cells and in intact preparations such as the optic nerve [100–102]. Dynamic modulation of astrocyte connectivity could therefore grade the spatial range of glial calcium signals, as well as tuning homeostatic roles such as metabolite redistribution and potassium buffering.

## 9. Information Processing by Glia and Computational Properties of Glial Plasticity

**Figure 1:**
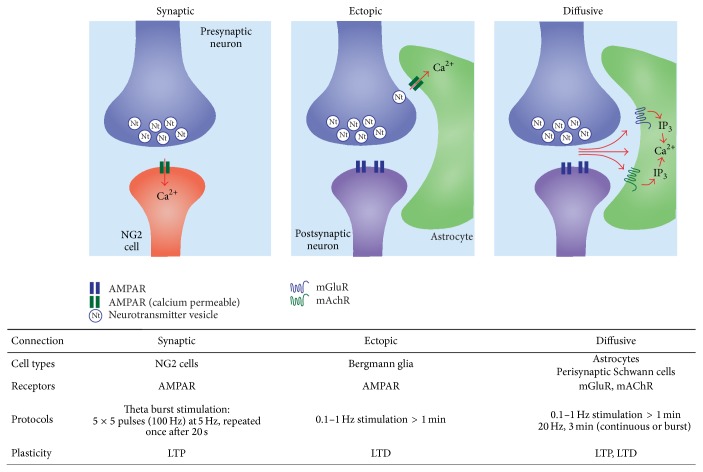
Modes of neuron-glial transmission and plasticity. Summary of the routes for neuron-glial transmission in which long-term plasticity after electrical stimulation of presynaptic cells has been demonstrated. Left panel shows synaptic transmission for NG2 cells (analogous to classical neuronal LTP), middle panel shows ectopic transmission at cerebellar Bergmann glia, and right panel shows volume transmission through diffusion to extrasynaptic receptors. The table shows cell types, receptors, stimulation protocols, and forms of glial plasticity that have been described. See main text for references and further details.

The accumulating evidence for neuron-glial plasticity indicates that signal processing through these routes for transmission can be altered in an activity-dependent manner, suggesting that memory processes could be accommodated within the network. However, it is striking that the computational properties of glial plasticity differ from those typical for induction of synaptic plasticity. The canonical example of synaptic plasticity is hippocampal LTP [2], which is typically evoked by a brief, high-frequency tetanus. Thus, a stereotyped incident signal is able to induce a change in synaptic strength within seconds, which can subsequently last for hours (or even years [8]). In contrast, plasticity appears to arise from less temporally precise induction signals (Figure 1).

For both the “priming” of mGluR responses in hippocampal neurons [49, 58] and the depression of AMPAR currents in Bergmann glia [72], plasticity requires prolonged repetitive stimulation at relatively low frequencies. For Bergmann glia, the immediate interval between stimuli has less impact on transmission strength than the average interval over several minutes [72]. A closely similar effect was observed in thalamic astrocytes, where calcium-dependent release of gliotransmitter increased only after long-term afferent stimulation, and was relatively insensitive to acute changes in transmission frequency [103]. These lines of evidence suggest that neuron-glial transmission is attuned to long-term trends in neuronal firing rates; in other words, astrocytes are sensitive to the average or integral of network activity rather than the detailed temporal structure of the spike train.

To date, most computational models of the role of astrocytes in network processing have focussed on gliotransmission at the level of the tripartite synapse [104]. Glia have been proposed to play a modulatory role that shapes short-term plasticity and the efficiency of transmission. These local feedback and feedforward roles may themselves be subject to graded modulation in response to average activity, which may result in long-term scaling of network connectivity.

In many respects these long-term shifts in glial sensitivity match well with the time frame over which information can feasibly be encoded into intracellular calcium oscillations. The frequency of spontaneous calcium signals observed in glia operates in the sub-Hz range [26, 85], and the duration of individual calcium “spikes” is typically in the tens of seconds range. If frequency modulation, mean interval, or variation in interval is exploited as encoding mechanism [104, 105], then the feasible window for input integration must typically extend over many hundreds or thousands of action potentials in the underlying network. Viewed from this perspective, release of gliotransmitters into the extracellular space may be a tonic pacing signal, which can be increased or decreased over a period of minutes in response to changes in average network activity. It is also interesting to note that probably the most convincing current example of a neurophysiological role for gliotransmission is in the control of sleep homeostasis [106]. Here glia release ATP that is metabolized to adenosine, providing a progressive drive of tonic adenosine that mediates a mounting sleep pressure after hours of wakefulness [107]. It certainly seems suggestive that neuron-glial plasticity is matched to temporal regimes that suit the longer lasting shaping of network activity that has been proposed as the clearest role for astrocytes in neurophysiology to date.

## 10. Future Directions

At present, the study of plasticity in neuron-glial transmission is in the discovery stage. Stimulation paradigms have been found that can cause lasting changes in glial responsiveness to neuronal activity, but we currently lack a deeper understanding of the molecular mechanisms underlying—and the detailed computational rules governing—induction, maintenance, and reversal of glial plasticity. Without this knowledge it will be difficult to hypothesize about feasible roles for glia in short-term information processing and long-term neurophysiological processes such as memory and learning.

A future goal to advance this understanding would be to determine the input-output relationship between synaptic stimulation and astrocyte calcium responses at what has been termed the tripartite synapse [108]. The fidelity and efficacy of transmission to the partner glial cell are likely to be markedly different from the postsynaptic neuronal partner and so will be governed by a different set of rules for translating a particular pattern of presynaptic firing into a particular pattern of extrasynaptic calcium responses (by analogy to an engineering system, neurons and glia are likely to have different transfer functions). Other goals would be more detailed understanding about the mechanisms controlling priming of astrocyte calcium responses during long-term stimulation, the mechanisms of crosstalk between signalling pathways, and the capacity for oligodendrocytes, microglia, tanycytes, and other glial cell types to express plasticity in their responses to neurotransmitters. A firmer foundation of understanding for these processes will undoubtedly help to refine and focus speculations on the potential active roles for glia in neurophysiology.
